# Supplementary figures and images for: Cooperation among c-subunits of FoF1-ATP synthase in rotation-coupled proton translocation
Source: eLife. 2022 Feb 2;11:e69096. doi: 10.7554/eLife.69096 (PMC8809890; doi:10.7554/eLife.69096)

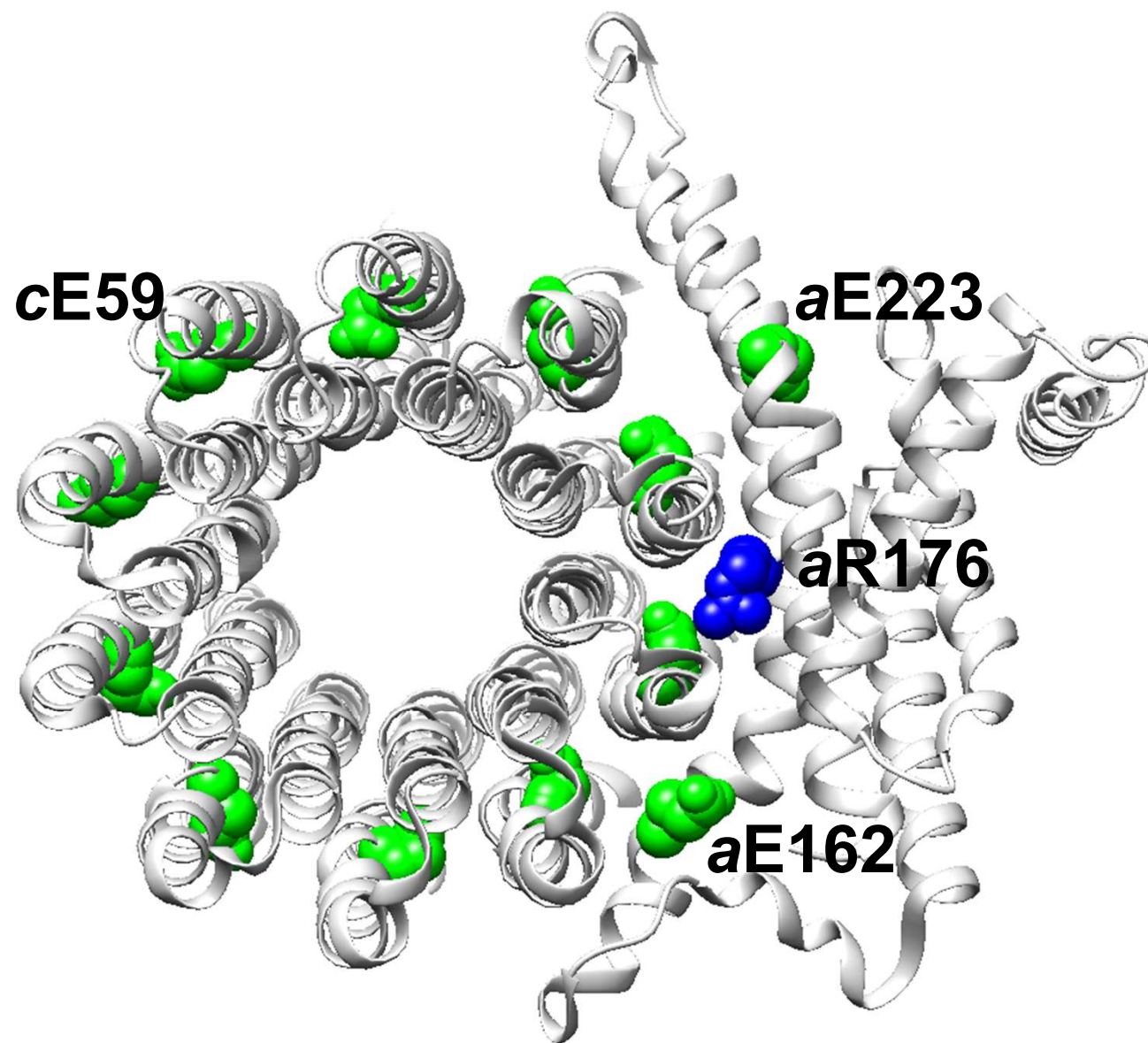

Supplement: Figure 1—source data 1. [file elife-69096-fig1-data1.pdf]

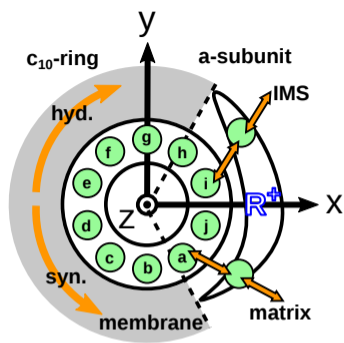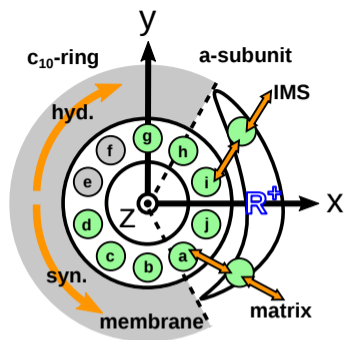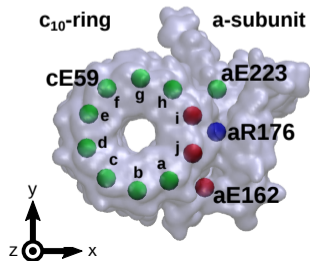

Supplement: Figure 1—source data 2. [file elife-69096-fig1-data2.pdf]

WT c<sub>10</sub> e ef eg eh ei ej

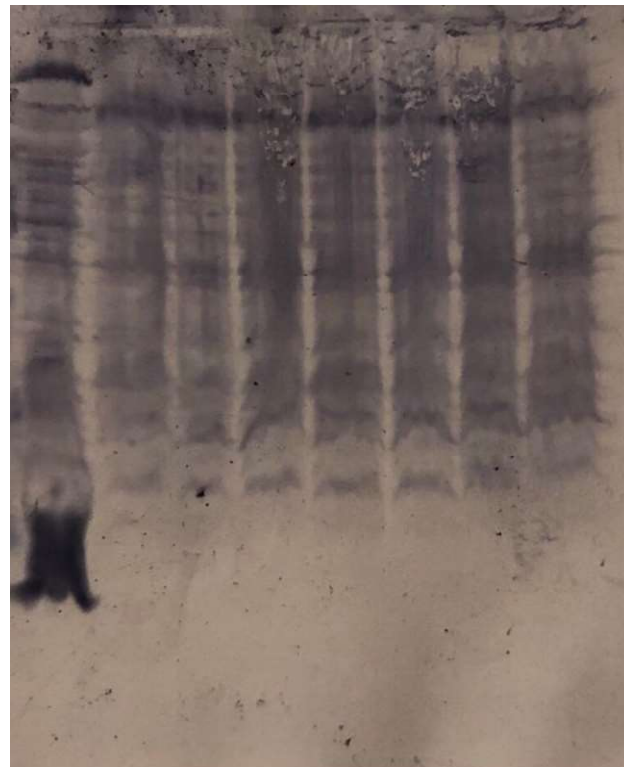

◀ C<sub>10</sub>

◀ C<sub>1</sub>

Supplement: Figure 2—source data 1. [file elife-69096-fig2-data1.zip › Figure 2-Sorce Data 2/Figure 2-Sorce Data 2.pdf]

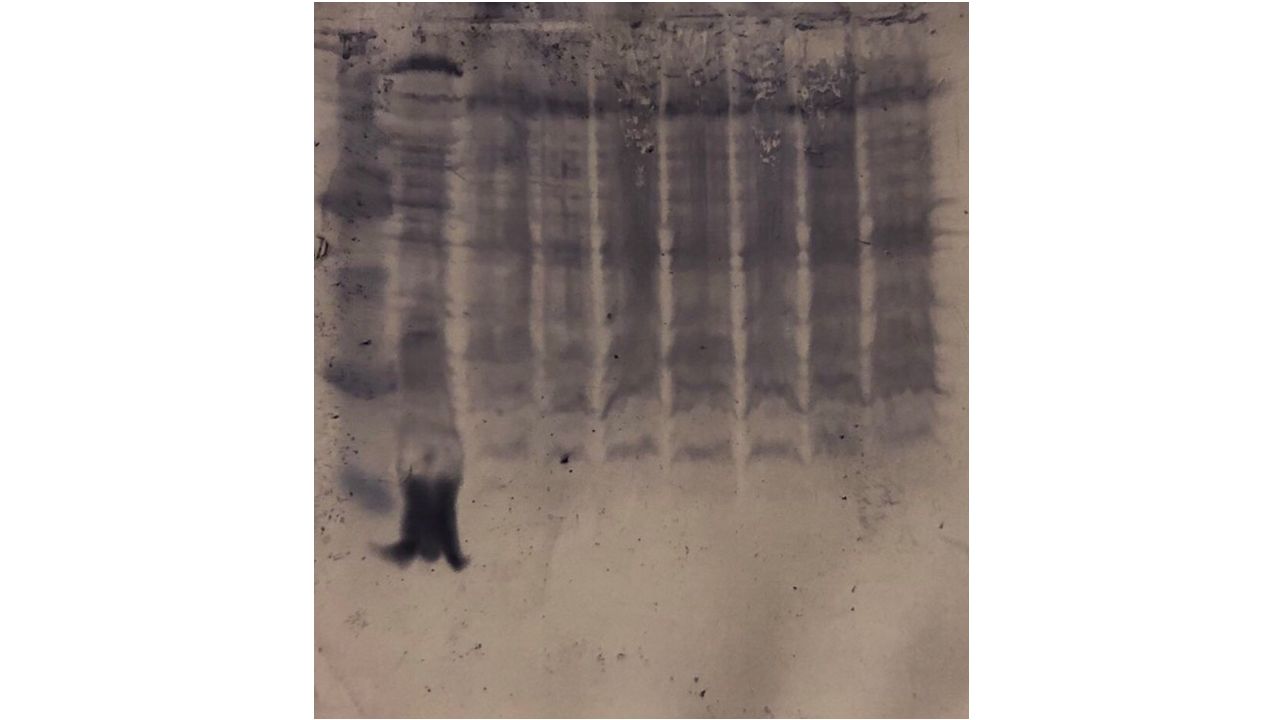

Supplement: Figure 2—source data 1. [file elife-69096-fig2-data1.zip › Figure 2-Sorce Data 2/Figure 2-Sorce Data 2.tif]
